# The practice of key essential nutrition action messages and associated factors among mothers of children from birth up to 2 years old in Wereilu Wereda, south Wollo zone, Amhara, Northeast Ethiopia: a community based cross-sectional study

**DOI:** 10.1186/s12887-019-1814-y

**Published:** 2019-11-25

**Authors:** Biruk Beletew, Bereket Gebremichael, Tewodros Tesfaye, Ayelign Mengesha, Mesfin Wudu

**Affiliations:** 1 Department of Nursing College of Health Sciences, Woldia University, P.O.Box 400, Woldia, Ethiopia; 2 School of Nursing and Midwifery, College of Health Sciences, Addis Ababa University, P.O.Box 400, Addis Ababa, Ethiopia

**Keywords:** Essential nutrition action, Practice, Mothers, Children, Wereilu, Ethiopia

## Abstract

**Background:**

The 1000 days, between a woman’s pregnancy and her child’s second birthday, offer a unique window of opportunity to build healthier and more prosperous societies. The right nutrition during this time can have a profound impact on a child’s ability to grow, learn, and rise out of poverty. An essential nutrition action is one of the most effective preventive actions for reducing nutrition-related morbidity and mortality among young children. Nonetheless, there is limited evidence regarding the practice of essential nutrition action and its associated factors.

**Objective:**

The main aim of this study was to assess the practice of key essential nutrition action messages and associated factors among mothers of children from birth up to 2 years old in Wereilu Wereda, South Wollo Zone, Northeast Ethiopia.

**Methods:**

A community-based cross-sectional study was conducted on 563 mothers of children from birth up to 2 years old in Wereilu Wereda from April 1–30, 2018. A multi-stage sampling technique was employed. A structured, adapted and pre-tested questionnaire was used to collect the data. The data was collected through the interviews. The data were entered in EPI-data Version 4.2 and exported to SPSS version 24.0 for analysis. Bivariate and multivariable logistic regression was carried out to asses’ associations between the outcome and independent variables. A *p*-value of < 0.05 was considered statistically significant in this study.

**Results:**

The prevalence of good practice was 256 (46.5%). Educational status of the mother and the father, monthly income, parity, place of birth, postnatal care follow-up, level of knowledge and level of attitude were predictors of good practice.

**Conclusion:**

The practices of key essential nutrition action messages in the study area were found to be low. So, it is better to improve access to information to the community on key essential nutrition action messages through trained health workers coupled with media.

## Background

Essential nutrition action (ENA) is an approach to expand the coverage of seven affordable and evidence-based nutrition actions to improve the nutritional status of women and children. It is an integrated package of preventive nutritional actions encompassing infant and young child feeding (IYCF), micronutrients and the nutrition of women [1, 2]. The seven ENAs are exclusive breastfeeding, complementary feeding, nutritional care of the sick children, nutrition for women during pregnancy and lactation, prevention of vitamin A deficiency, prevention of anemia, and prevention of iodine deficiency [3]. Pregnancy to the age of 24 months of a child is the critical window of opportunity for the delivery of essential nutrition interventions [4]. Universal coverage of essential nutrition actions could prevent more than 2 million mothers and child death each year [5]. Children who get the right nutrition in their first 1000 days are 10 times more likely to overcome life-threatening childhood diseases and have healthier families of their own [3].

The poor practice of seven essential nutrition actions contributes to about one-fourth (25%) of nutrition-related morbidity and mortality rates [2, 6]. Unfortunately, more than half of the world’s children do not have access to these lifesaving actions [2]. Sub-optimal IYCF practices alone increase the risk of infant and child morbidity and mortality by up to five-fold. Maternal under-nutrition is estimated to account for 20% of childhood stunting [4]. Many children are born undernourished because their mothers are undernourished and as many as 50% of all children become stunted utero [5]. When pregnant women are provided with balanced nutrition there is approximately 31% reduction in giving birth to children who are small gestational age (SGA) [6].

The 2016 Ethiopia Demographic Health Survey (EDHS) report revealed that 38% of under 5 children are stunted while 18% of them are severely stunted [7]. In Ethiopia, the IYCF practice remains below the WHO standard; only 58% of infants exclusively breastfed and only 7% of young children given optimal complementary feeding [8]. Nine percent of infants less than 6 months of age use a bottle to feed [7]. Among African countries, Ethiopia has the largest burden of micronutrients deficiency; 39.9% of children in Ethiopia are iodine deficient [9]. Only 23.3% of households used adequately iodized salt [10]. In Ethiopia, one in every six women in the reproductive age group (17%) is anemic [11, 12]. An estimated 33.9% of children up to age 2 are vitamin A deficient in Ethiopia [13]. Furthermore, in the Amhara region, the ideal feeding practice is low where the practice of timely initiation of breast feeding (TIBF), minimum meal frequency (MMF), and minimum dietary diversity (MDD) being 38, 34 and 2.1% respectively [12]. The prevalence of stunting in the Amhara region was high (46%) [14]. Poor practice of ENA has contributed to a significant prevalence of malnutrition among children. If implemented effectively, the key ENA messages could reduce nutrition-related mortality and disease burden by 25% [2]. Thus, it is critical to break the inter-generational cycle of malnutrition by implementing the seven essential nutrition actions during the first 1000 days [1]. Therefore this study was aimed to asses’ the practice of key ENA messages and associated factors among mothers of children from birth up to 2 years old age.

## Methods

### Study area and period

The study was conducted in Wereilu Wereda from April 1–30, 2018. Wereilu is one of the Wereda in South Wollo Zone of Amhara Region which is found at 492 km far from Addis Ababa, Northeast Ethiopia. The total population of Wereilu is 127,041. The Woreda has a total of 24 kebeles (neighborhood units); among which 20 kebeles are rural and 4 kebeles are urban. In Wereilu Wereda there are 5 health centers and 1district hospital. The number of children from birth to 24 months old in this Wereda is 6426 [15].

### Study design

A community-based cross-sectional study design was employed.

### Population

#### Source population

Mothers of children aged from birth up to 2 years old in Wereilu Wereda.

#### Study population

Mothers of children aged from birth up to 2 years old in selected kebeles of Wereilu Wereda. The age range of the mothers who participated in the study was from 17 to 47 years old.

### Eligibility criteria

#### Inclusion criteria

All mothers who have children aged between births up to 2 years old and live in Wereilu Wereda for more than 6 months were included in the study.

#### Exclusion criteria

Mothers who were not able to speak or who were severely ill (due to different problems) were excluded from the study.

### Sample size and sampling procedure

#### Sample size determination

The sample size was calculated using the single population proportion formula considering the proportion (P) 56.5% from the previous study [16], level of confidence 95%, and margin of error 5%. Since the size of the source population was finite population (less than 10,000) which is 6426 correction formula was applied. After determining the sample size a design effect of 1.5 and 5% non-response rate was used. The final calculated sample size was 563.

#### Sampling procedure

From the total 24 kebeles (4 urban and 20 rural) 7 rural and 2 urban kebeles were selected by using a simple random sampling method. The samples were distributed proportionally based on probability proportional to size (PPS) allocation technique. Participants in each kebeles were selected by using a systematic sampling technique after calculating the sampling interval (K) for each kebeles. In house-holds with two children less than 2 years of age, one was selected randomly by lottery method. Homes were revisited three times when eligible respondents were not available by the time of the survey. Those who were not available after revisit were considered as non-respondent.

#### Operational definitions

##### Essential nutrition action (ENA):

Is an integrated package of preventive nutritional actions encompassing: exclusive breastfeeding, complementary feeding, nutritional care of the sick children, nutrition for women during pregnancy and lactation, prevention of vitamin A deficiency, prevention of anemia, and prevention of iodine deficiency.

##### Good ENA practice:

Those respondents who score mean and above mean score of practice questions.

##### Poor ENA practice:

Those respondents who scored below mean score of practice questions.

##### Kebele:

A kebele is the smallest administrative unit of Ethiopia. It is a part of Wereda (District), itself usually part of Zone, which in turn are grouped into one of the regions based on Ethno-linguistic communities that comprise the Federal Democratic Republic of Ethiopia.

##### Exclusive breastfeeding (EBF):

Is giving only breast milk to infants up to 6 months old allowing only ORS drops syrups or medicines.

##### Complementary feeding:

Initiation of additional solid or semi-solid or soft foods along with breast milk starting from 6 months.

### Data collection tool and procedure

Data was collected by a pretested, structured, interviewer-administered questionnaire developed by the Food and Agriculture Organization publication guidelines for assessing nutrition-related knowledge, attitudes and practices (2014), also called KAP manual [17]. The questionnaire had 9 parts: Part1- Socio-demographic /economic variables, Part 2- questions on exclusive breastfeeding, Part 3- questions on complementary feeding, Part 4- questions on feeding of sick child, Part 5- questions on nutrition during pregnancy and lactation, Part 6- questions on prevention of Vitamin A deficiency, Part 7- questions on prevention of anemia (10 questions), Part 8-KAP questions on prevention of Iodine and Part 9 – questions which asses access for nutritional information (Aditional file 1). Those respondents who score at the mean or above were classified as having a good practice and those who score below mean were classified as having poor practice. The questionnaire was first developed in English and translated to the Amharic language by an expert. It was back-translated to English by an independent translator to check for consistency. Interview was conducted with mothers of the index child at their home to ensure privacy. Seven diploma nurses and 2 BSc degree nurses were recruited as data collectors and supervisors respectively.

### Data quality control

The data collectors and the supervisors were trained for 2 days on the techniques of data collection and the importance of disclosing the possible purposes of the study to the study participants before the start of data collection. To assure the quality of the data, supervisors, and the investigators closely supervised the data collection procedures daily. A review and correction was conducted in the field to check the completeness of questionnaire. Each questionnaire and data sheet was checked before the data entry. The data were entered daily and missing data were identified. Questionnaire containing errors were excluded from the study.

A pretest was conducted in Jamma district (which is not a study area) using 56 women who were not included in the actual sampling and necessary adjustments were made on the tool. Jamma district is situated in a neighboring area of Wereilu district where the population shares similar attributes with that of the Wereilu district population.

### Data processing, analysis, and presentation

Data were entered into statistical software EPI-data Version 4.2 upon creating the questionnaire template. The entered data were subjected to cleaning using simple frequency and tabulation to ensure the validity of the data. Then the analysis was conducted with IBM SPSS version 24 after exporting the prepared data. Descriptive statistics like frequency, proportions, and percentage were computed. Bivariate and multivariable logistic regression was performed to examine the associations between dependent and independent variables. Those variables that had *p*-values ≤0.25 were included in the multivariable logistic regression model to adjust for possible confounders. *P*-value < 0.05 was considered statistically significant in this study.

## Result

### Socio-demographic characteristics of study participants

Out of 563 mothers who have children from birth up to 2 years old who were selected to participate, 550 took part in the study with a response rate of 97.7%. About 213 (38.7%) of the mothers were within the age range from 30 to 34 years. In terms of their family size, most of the respondents, 233(42.3%) had 4–5 children. Only 53 (9.7%) respondents were an employed. Concerning the children’s characteristics; more than one third (37.5%) of children’s age was from 6 months to 12 months. In addition, 390 (70.9%) respondents delivered their child at the health institution. About 303 (55.1%) and 247 (44.9%) of the respondents have antenatal care (ANC) and post natal care (PNC) follow up respectively (Table 1).

**Table 1:** Demographics and related characteristics of mothers of children from birth up to 2 years old in Woreilu Wereda, South Wollo Zone, Amhara, Ethiopia, 2018 (*N* = 550)

Variables/ characteristics	N (%)	The practice of key ENA messages		Odds Ratios		*P*-value
		Good	Poor			
		Frequency (%) 256(46.5)	Frequency (%) 294(53.5)	COR(95% CI)	AOR(95% CI)	
Age of the mother						
< 20 years	11(2.0)	5(0.9)	6(1.1)	1.25(.74–1.98)	1.12(.21–1.82)	.75
20–24 years	99(18.0)	43(7.8)	56(10.2)	1.15(0.74–1.66)	.76(.23–1.93)	.55
25–29 years	187(34.0)	88(16)	99(18)	1.33(.65–1.98)	.90(.51–1.62)	.34
30–34 years	213(38.7)	104(18.9)	109(19.8)	1.43(.56–12.04)	1.28(.52–1.32)	.23
> 35	40(7.3)	16(2,9)	24(4.4)	1	1	
Ethnicity						
Amhara	522(94.9)	245(44.5)	277(50.4)	0.90(.40–2.51)		
Oromo	17(3.1)	6(1.1)	11(2)	0.54(.30–1.11)		
Tigre	9(1.6)	4(0.7)	5(0.9)	0.8(.40–1.93)		
Others	2(0.4)	1(0.2)	1(0.2)	1		
Religion						
Orthodox	224(40.7)	109(19.8)	115(20.9)	1.42(.55–11.24)	1.22(.51–1.82)	.65
Protestant	148(26.9)	65(11.8)	83(15.1)	1.17(0.68–1.76)	.76(.28–1.98)	.35
Muslim	173(31.5)	80(14.5)	93(16.9)	1.30(.55–1.92)	.90(.57–1.82)	.43
Catholic	5(0.9)	2(0.4)	3(0.5)	1	1	
Marital status						
Single	39(7.1)	16(2.9)	23(4.2)	1.32(.56–1.98)	1.33(.31–1.72)	.22
Married	482(87.6)	230(41.8)	252(45.8)	1.73(.05–9.24)	.66(.37–1.88)	.45
Separated /Divorced	29(5.3)	10(1.8)	19(3.5)	1	1	
Educational status of the mother						
Illiterate	287(51.25)	85(33.2)	202(68.7)	1	1	
Primary	162(28.93)	102(39.8)	60(20.4)	4(2.7–6.07)^**^	2.7(1.55–4.9)^**^	.001
Secondary and above	101(18.04)	69(27.0)	32(10.9	5(3.14–8.36)^**^	3.98(1.93–8.22)^**^	<.001
Educational status of the father						
Illiterate	341(60.89)	111(43.4)	230(78.2)	1	1	
Primary	131(23.40)	88(34.4)	43(14.6)	4(2.76–6.514)^*^	2.02(1.14–3.57)^*^	.015
Secondary and above	78(13.93)	57 (22.3)	21(7.1)	5(3.247–9.74)^**^	2.784(1.35–5.719)^**^	.005
Occupation of the mother						
Not employed	262(47.6)	126(22.9)	136(24.7)	1.85(.15–10.24)	.60(.2–1.40)	.17
Housewife	218(39.6)	103(18.7)	115(20.9)	1.79((.02–8.94)	.711(.25–1.64)	.26
Governmental Employee	52(9.5)	21(3.8)	31(5.6)	1.35(.57–1.93)	.61(.305–1.224)	.165
Employed in private sector	18(3.3)	6(1.1)	12(0.2)	1	1	
Residence						
Urban	115(20.54)	79(30.9)	36(12.2)	3.2(2.06–4.96)^*^	.908(.51–1.62)	.744
Rural	375(66.9)	117(69.1)	258(87.8)	1	1	
Monthly income						
Lower	304(86.68)	104(40.6)	200 (20.4)	1	1	
Medium	168(30)	134(52.3)	34(68.0)	1.26(.68–2.3)^*^	2.2(1.03–4.8)^*^	.041
Higher	78(13.93)	18(7.0)	60(11.6)	3.27(1.7–6.3)^**^	4.4(1.9–10.2)^**^	.001
Number of family member						
< 4	106(19.3)	50(9.1)	56(10.2)	1.09(.88–1.89)		
4–5	233(42.4)	111(20.2)	122(22.2)	1.11(.67–1.95)		
6–8	122(22.2)	55(10)	67(12.2)	1.01(.67–1.43)		
≥ 9	89(16.2)	40(7.3)	49(8.9)	1	1	
Parity						
Multi-para	427(76.25)	218(85.2)	209(71.1)	2.3(1.5–3.57)^*^	2.1(1.17–3.78)^*^	.013
Primi-para	123(21.96)	38(14.8)	85(28.9)	1	1	
Age of the child						
< 6 month	181(32.9)	83(15.1)	98(17.8)	1.08(.78–1.79)		
6–12 month	206(37.5)	98(17.8)	108(19.6)	1.16(.67–1.83)		
12–18 month	106(19.3)	50(9.1)	56(10.2)	1.14(.77–1.97)		
18–24 month	57(10.3)	25(4.5)	32(5.8)	1		
Sex of the child						
Male	324(57.85)	164(64.1)	160(54.4)	1.49(1.06–2.1)^*^	1.18(0.74–1.86)	.480
Female	226(40.36)	92(35.9)	134(45.6)	1	1	
Place of birth						
Health institution	390(69.64)	194 (75.8)	196(66.7)	1.56(1.07–2.27)^**^	2.95(1.69–5.16)^**^	<.001
Home	160(28.57)	62(24.2)	98(33.3)	1	1	
ANC follow-up						
Yes	303(54.11)	186(72.7)	117(39.8)	4.02(2.8–5.7)^*^	1.2(.74–1.96)	.458
No	247(44.11)	70(27.3)	177(60.2)	1	1	
PNC follow-up						
Yes	247(44.11)	156(60.9)	91 (31.0)	3.48(2.45–4.95)^**^	2.17(1.35–3.49)^**^	.001
No	303(54.11)	100 (39.1)	203 (69.0)	1	1	
Heard nutritional related information						
Yes	347(61.96)	230(89.8)	177(73.8)	3.14(1.94–5.08)^*^	.611(.305–1.224)	.165
No	143(25.54)	26(10.2)	117(26.2)	1	1	
Knowledge level						
Good	365(65.18)	222(86.7)	143(48.6)	6(4.49–10.57)^**^	3.2(1.86–5.58)^**^	<.001
Poor	185(33.04)	34(13.3)	151(51.4)			
Attitude level						
Good	379(67.68)	233(91.0)	146(49.7)	10(6.32–16.68)^**^	5.5(3.14–9.8)^**^	<.001
Poor	171(30.53)	23(9.0)	148(50.3)	1	1	

*indicates P value < 0.05

**indicates P value < 0.01

### The practice of key essential nutrition action messages

Out of 550 study participants who responded to the questions to assess practices of key ENA messages, 256 (46.5%) respondents had a good practice of key ENA messages.

### Factors associated with the practice of key essential nutrition action messages

In bivariate logistic regression educational status of the mother and the father, monthly income, parity, sex of the child, place of birth, having ANC follow up, having PNC follow up, level of knowledge and level of attitude were significantly associated with the practice of key ENA messages. Those variables that have *p*-value ≤0.25 were taken to the multivariable logistic regression model to adjust for possible confounders.

In multivariate logistic regression educational status of the mother and the father, monthly income, parity, place of birth, PNC, level of knowledge and level of attitude were significantly associated with the practice of key ENA messages. Accordingly, the odds of good practice of key ENA messages among children whose mothers attended primary school [AOR = 2.7,95% CI: (1.548–4.905)] and secondary school and above [AOR = 3.9, 95% CI: (1.929, 8.217)] was higher as compared to those illiterate mothers. Similarly, the odds of the practice of key ENA messages was higher among fathers whose educational level was secondary and above [AOR = 2.7, 95% CI: (1.356–5.719)] as compared to those fathers who were illiterate. The odds of the practice of key ENA messages was higher among fathers whose educational level was primary (AOR = 2.0, 95% CI: (1.145–3.577)) as compared to those fathers who were illiterate. Monthly income was also associated with the practice of key ENA messages. Those who have higher monthly income were 4.4 times more likely to have a good practice of key ENA messages than those who have lower income. Those who have medium income were also 2.2 times more likely to have a good practice of key ENA messages than those who have lower income. Regarding parity children of multipara mothers were 2.1 times more likely to have a good practice of key ENA messages than children of primipara mothers. Concerning the place of birth, the odds of good practice of key ENA messages among children who have been delivered in a health institution (AOR = 2.9, 95% CI: (1.692–5.157)) was higher as compared to those who have been delivered in their home. Furthermore, mothers who had PNC follow-up were 2.2 times more likely to practice the key ENA messages than those mothers who did not have PNC follow-up. Besides, mothers who have good knowledge were 3.2 times more likely to practice the key ENA messages than those with poor knowledge. Moreover, increased odds of the practice of key ENA messages were noted among mothers who had a good level of attitude towards key ENA messages [AOR = 5.5,95% CI: (3.146–9.8)] compared to those who had the poor attitude (Table 1).

### The practice of exclusive breastfeeding

Almost all, 544 (98.7%) of the respondents had ever breastfed their children. About 464 (84.2%) respondents initiate breastfeeding immediately after birth within 1 h. One hundred forty-nine (82.3%) infants less than 6 months were exclusively breastfed. About 192 (34.9%) respondents consume the pre-lacteal feed. About 209 (38%) of the respondents squeeze out and threw away the first milk (colostrum). Regarding bottle feeding 272 (49.5%) of the respondent’s children drink from a bottle (Table 2).

**Table 2:** Practice of EBF among mothers of children from birth up to 2 years old in Wereilu District, South Wollo Zone, Ethiopia, 2018

Variable		Frequency	Percent
Ever breastfed	Yes	544	98.9
	No	6	1.1
Initiation of breastfeeding	Immediately	464	84.4
	Hours after birth	76	13.8
	Days after birth	10	1.6
Exclusive breastfeeding	Yes	149	82.3
	No	32	17.7
Pre-lacteal feed	Yes	192	34.9
	No	358	65.1
Squeeze out colostrum	Yes	209	38.0
	No	341	62.0
Bottle feeding	Yes	272	49.5
	No	278	50.5

### The practice of respondents on complementary feeding

Among 206 mothers with children aged between 6 and 12 months old, 197 (95.6%) of them started giving complementary feeding for their infants. About 130 (63.5%) of mothers of children from 6 months − 12 months introduced liquid or semi-solid foods at 6 months. Thirty-nine (18.8%) respondents started complementary feeding after sixth months and thirty-six (17.7%) of children were initiated before they reached the sixth month. Concerning minimum meal frequency out of 369 mothers of children from 6 months up to 2 years old below half, 162 (44%) of mothers of children from 6 months up to 12 months give their child complementary foods 3 times per day. The majority, 314 (85%) of the children had poor dietary diversity. Among 106 mothers who had 12–18 months old children, 86 (81.1%) of them continued to breastfeed their children at 1 year. Among 54 mothers who had children aged from 20 to 24 months, 42 (77.8%) of them continued to breastfed at age 2 years.

### The practice of feeding the sick child during and after illness

Among the total 550 participants, 202 (36.5%) participant’s children were sick in the last 2 weeks; of those, only 40 (19.8%), 85 (42.1%) and 77 (38.1%) mothers of sick children gave more than the usual amount of breastfeeding, fluid, and food respectively. About 69.2, 43.6, and 47% of respondents gave the same amount of breastfeeding, fluid, and food respectively. The rest 0.5 and 2% of respondents gave less than the usual amount of fluid and food respectively.

### The practice of mothers on the nutrition of women’s during pregnancy and lactation

Four hundred sixty-nine (85.3%) respondents practiced the habit of eating snacks during their pregnancy and lactation period. About 437(79.5%) respondents ate a variety of foods, particularly animal products (meat, milk, egg) plus fruit and vegetables during pregnancy and lactation. Four hundred seven (74%) respondents have got an iron/folic acid pill during pregnancy while 121(22%) did not take iron/folic acid pill during and the rest 22(4%) doesn’t remember whether they had taken or not.

### The practice of mothers on vitamin a prevention

Three hundred forty-nine (63.5%) respondents have taken vitamin A supplementation within 45 days after delivery. About 255(69.1%) respondent’s children had taken vitamin A supplementation 2 times a year after 6 months of age. One hundred sixty-two (29.5%) participants eat animal-source foods-liver, kidney, heart, and egg yolks/egg from the chicken, Milk, cheese, yogurt or other dairy product within the last 24 h. One hundred seventy-eight (32.4%) participants eat green vegetables-Amaranths, spinach, cassava leaves, kale, and other green leafy vegetables with in the last 24 h. Two hundred sixty-five (48.2%) respondents eat Fruits-Ripe mango, ripe papaya within the last 24 h.

### The practice of mothers on prevention of anemia

Four hundred seven (74%) mothers received iron or folic acid during pregnancy; the rest 143 (26%) respondents did not. Four-hundred fifty-three (82.4%) respondents eat meat and animal products during pregnancy. Four-hundred forty (80%) participants eat green leafy vegetables and fruit during pregnancy; the rest 110(20%) participants did not eat.

### The practice of mothers on prevention of iodine deficiency

Four hundred fifty-six (82.9%) respondents used iodized salt while they cook family food; However, 94 (17.1%) of the respondents used non-iodized salt. Four hundred four (73.5%) respondents did add salt to stew at the end, while the rest 137(24.9%) added salt at middle and 9(1.6%) added salt at the beginning. About 483 (87.8%) respondents did store salt in dark closed containers; the rest 67(12.2%) did not.

### Access to nutritional related information

Regarding nutrition-related information 416 (75.6%) respondents heard a message about maternal infant and young children feeding practice. Three hundred three (33.7%) respondents had the information from nurses followed by 277(30.8%) from Health Extension Workers; another source of information is from media 116(12.9%), midwives 115(12.8%) and doctors 88(9.8%). Three hundred eighty-nine (44.7%) respondents had the information during pregnancy followed by during their post-natal period 186(21.4%); the rest 130(14.9%), 82(9.4%), 48(5.5%), and 35(4%) had nutritional information during delivery, immunization, sick child visit, and well-child visit respectively. Regarding where the information was given 440(57.9%) respondents have got at heath facility, 246 (32.4%) in their home and 74 (9.7%) during community events.

## Discussion

This study was performed to assess the practice of key ENA messages and associated factors among mothers of children from birth to 2 years old in Wereilu Wereda. Accordingly, the overall prevalence of good practice was 46.5%.

This study revealed that 82.3% EBF practice which is almost consistent with a study in Bahirdar [18]. However, this practice is higher than the study finding in Azezo District [19], in the Oromia Region [20], and Al Hassa-Saudi Arabia [21]. Possible justifications for this variation could be the increased maternal and child health service utilization in this study such as the use of delivering at a healthcare facility (70.9%).In this study, 84.2% of mothers initiated breastfeeding within the first hour after delivery. This finding is higher than previous study findings in Tanzania [22] and in India [23]. The discrepancy might be due to the number of mothers who had ANC follow up and who give birth at a health institution is dramatically increasing due to the persistent promotion of the free delivery service provider in the country. As per this study, 49.5% of mothers used a bottle to feed their index child which is not a WHO recommendation. This finding is higher than the previous study finding in Bahirdar [18], in India [24]. In this study 192 (34.9%) mothers gave pre-lacteal feeds to their index child; This finding is higher than EDHS 2011[25], in Bahirdar [18]. This might be explained by the majority of mothers in the current study had no formal education and a lack of access to mass media because of their poor socioeconomic status and most of the respondents of this study were from the rural area, where there is less access to nutrition-related information.

Regarding complementary feeding, 95.6% of mothers of children from 6 months to 1 year started giving complementary foods or drinks. This finding is consistent with the study finding in Bahirdar [18], Nairobi, Kenya [26]. However, this finding is higher than the previous study finding in Ethiopia [27] and India [24]. The difference might be due to the governmental and non-governmental organizations that are currently promoting the benefit of complementary feeding through professionals and mass media. TICF in this study was 63.5% which is almost consistent with a study in Northwest Ethiopia, 61.5% [28] and in Lalibela [16]. This might be explained by similarity in the lifestyle, socioeconomic status, and cultural factors. However, this finding was lower than the WHO cut-off point (80 to 94%) for good practice of complementary feeding [16] and it was higher than study in Nigeria 57% [29] and 39.2% in Pakistan [30], 55% in India [31]. Possible justifications for this discrepancy might be Health extension workers are making home to home visits on regular bases to support families in accessing basic health services and to give home-based health education as well as other promotion services, including the promotion of appropriate IYCF. According to this study, about 74% of 6–23 months old children have been given the minimum meal frequency. This finding is higher than the study findings in Northwest Ethiopia [28], India [32] and Pakistan [33]. Additionally, 15% of children aged between 6 and 23 months old have received minimum meal diversity. This finding is higher than the study findings in Ethiopia [18] and Pakistan [33]. One possible explanation for this discrepancy might be the improvements in nutrition education and counseling in the community as components of maternal and child health care services.

The majority of mothers in the sample (87.4 and 81.1%) of mothers have continued to breastfeed their children at age one and 2 years respectively. This finding is in line with a previous study finding in Ethiopia [34]. These high rate might be because to the majority of women participated in these studies were housewives and spend much of their time at home a factor known to the likelihood of continuing to breastfeed. Only 38.1% of mothers who have sick children increase the frequency of feeding during illness. This finding is higher than research in India 11.5% [35]. A possible explanation for this might be in Ethiopia health extension workers are making home to home visits on regular basis to support families in accessing basic health services and to give home-based health education as well as other promotion services.

Regarding adequate nutrition for women during pregnancy and lactation, this study revealed that 85.3% of the respondents had practiced the habit of eating snacks during their pregnancy and lactation period. This finding is higher than the study finding in East Wellega Zone, 59.9% [36]. According to this study, 74% of respondents have got an iron/folic acid pill during pregnancy which is higher than finding in India 62% [37]. The reason for this discrepancy might be explained by the improvements in the utilization of ANC and institutional delivery.

Concerning the prevention of vitamin A deficiency in this study, 162(29.5%) participants eat vitamin A-rich foods on the day preceding the survey; which was higher than the study from Sidama 25.6% [38]. According to this study, 63.5% of respondents have taken vitamin A supplementation (immediately) within 45 days after delivery. Which is higher than study in Ethiopia 10.7% [39]. This discrepancy might be due to the difference in the socio-cultural status of the participants and improvement in the implementation of maternal and child nutrition-related strategies.

Regarding the prevention of anemia, this study showed that 82.4% of respondents eat meat and animal product during pregnancy; 80% of the participants eat green leafy vegetables and fruit during pregnancy. These results are comparable with the previous study in the Amhara region [40]. This might be due to the similarity in the socio-demographic characteristics of the respondents.

Concerning the prevention of iodine deficiency according to this study, 82.9% of respondents used iodized salt while they cook family food. This finding is higher than the study finding in Ghana 64.6% [41]. In this study, most of, 87.8% of respondents did store salt in dark closed containers. This is higher than study finding in Ghana 62.6% [41] and Burnie and Womberma Districts, Ethiopia [42]. The probable reasons for this discrepancy could be a variation in the study period and the increased effort made in Ethiopia to achieve the nutritional strategies through health extension workers.

Regarding factors associated with the practice of key ENA messages, the result of the multivariate analysis showed that the odds of the practice of key ENA messages were higher among mothers whose educational level were secondary and above [AOR = 3.98, 95% CI: 1.929, 8.217) as compared to that of illiterate mothers. Parallel findings were reported by studies in Northern Ethiopia (AOR = 3.84) [43],in Lasta [AOR = 9.50; 95% CI:1.02, 14.25] [16] and in Bahirdar (AOR 3.0; 95% CI 1.2, 8.6) [18]. This might be because educated mothers have a better understanding of nutrition education than less-educated mothers. Additionally, educated mothers might obtain information from different sources like; books, leaflets and magazines, and might have a better chance of exposure to nutrition education through mass media than their counterparts. Similarly, the odds of the practice of key ENA messages were higher among fathers whose educational level was secondary and above [AOR = 2.78, 95% CI: 1.356, 5.719] and primary level (AOR = 2.02, 95% CI: 1.145, 3.577) as compared to those fathers who were illiterate. This could be because fathers’ knowledge regarding key ENA messages are affected by their education level and that is ultimately determines maternal practice on optimal maternal and child nutrition and health-seeking behavior. The current study revealed higher odds of key ENA message practice among mothers who delivered in health institutions as compared to those who delivered in their home. This finding was in line with research conducted in Azezo District (AOR 2.18; 95% CI 1.22, 4.35) [19]. This could be explained as mothers who give birth at health institutions have a better opportunity to access appropriate maternal and child feeding information, which ultimately decreases exposure to traditional beliefs immediately after birth. Moreover, a significant association was observed between the practice of key ENA messages and attending postnatal care service (AOR = 2.177,95%CI:1.356, 3.496). This finding is consistent with previous study findings in Bahirdar (AOR 4.1; 95% CI 1.4, 12.2) [18],in Lasta (AOR = 5.98; 95% CI:1.49, 13.96) [16], and Northern Ethiopia (AOR = 2.80) [13]. This might be due to the result of information and counseling that the mothers received from health workers during their postnatal visits. Income was also significantly associated with the practice of key ENA messages (AOR = 4.397; CI:1.899, 10.182). It is also supported by evidences from Azezo District (0.25 (0.15, 0.49) [19] and in North West Ethiopia (AOR = 2.18, CI: 1.39, 3.39 [44]. A Possible justification for this could be families with high income can pay the requested fee to utilize MCH services and they can also easily access and implement nutrition-related information. Multipara mothers had more practice of key ENA messages (AOR =2.106; 95%CI: 1.172, 3.786) than primipara mothers. The reason for this could be that as the parity increased, infant management experiences will also be increased. Furthermore, exposure of mothers to nutritional related information was found to be significantly associated with the good knowledge level of key ENA messages (AOR = 1.999, CI: 1.142, 3.499). About respondents’ level of knowledge, the odds of the practice of key ENA messages were higher among mothers with high knowledge of key ENA messages (AOR = 3.224; 95%CI: 1.86, 5.588) as compared to their counterparts with a lower-key ENA message knowledge. Besides, mothers with a good level of attitude towards key ENA messages were 5.55 times more likely to have a good practice of key ENA messages. The finding was supported by another study report elsewhere in Northwest Ethiopia [27]. This shows that boosting mothers’ knowledge of key ENA messages and changing the attitude of mothers towards key ENA messages is the cornerstone for implementing sustainable strategies to improve appropriate ENA practices [45]. However, there was also a gap between the knowledge (46.5%) and attitude (66.4%) of mothers with their practices (68.9%). This might indicate the presence of some cultural practice that could affect the practice of key ENA messages and poor socioeconomic status to implement the recommendations they know or pressure by another part of the family on the mothers.

## Limitations

The cross-sectional nature of this study makes causal relationships between dependent and independent variables impossible. The response may have been affected by social desirability and recall bias.

## Conclusion

This study provided a baseline finding on the status of the practice of key ENA messages in the study area. The practices of key ENA messages in the study area were found to be low. Higher educational status of the mother and the father, higher monthly income, multiparty, institutional delivery, having PNC follow -up, level of knowledge and level of attitude were significantly associated with the good practice of key ENA messages.

### Recommendation

Based on the finding of this study, the following recommendations were developed. Training of health workers on ENA recommendation guidelines should be used to enhance their knowledge and skills; better trained health workers may be more likely to transfer health information for mothers at health institutions and in the community. Current health education efforts should be strengthened to stepping-up MCH service utilization by focusing on uneducated and rural women. Media have to promote social behavioral change and communication related to key ENA messages. Future studies should emphasize mixed methods, such as triangulating with a qualitative study design and prospective study designs to identify factors for the practice of key ENA messages.

## Supplementary information

**Additional file 1.** QuestionnaireR4.

## Data Availability

Data will be available upon a reasonable request made to the correspondent author.

## Abbreviations

ANC: Antenatal Care
EBF: Exclusive Breast Feeding
EDHS: Ethiopia Demographic Health Survey
ENA: Essential Nutrition Actions
IYCF: Infant and Young Child Feeding
MCH: Maternal Child Health
PNC: Post Natal Care
TIBF: Timely Initiation of Breast Feeding
TICF: Timely Initiation of Complementary Feeding
UNICEF: United Nation Children Fund
USAID: United States Agency of International Development
