# Supplementary material for: The practice of key essential nutrition action messages and associated factors among mothers of children from birth up to 2 years old in Wereilu Wereda, south Wollo zone, Amhara, Northeast Ethiopia: a community based cross-sectional study
Source: BMC Pediatr. 2019 Nov 25;19:459. doi: 10.1186/s12887-019-1814-y (PMC6876089; doi:10.1186/s12887-019-1814-y)
Supplement: Supplementary file 1 — Additional file 1. QuestionnaireR4. [file 12887_2019_1814_MOESM1_ESM.docx]

**Annexe 1: Information sheet**

1. **English version**

Addis Ababa University, School of allied health sciences, Department of Nursing and Midwifery

**INTRODUCTION**: Good morning/afternoon? My name is ____________________. In this study which is undertaken by Addis Ababa University, College of Health sciences, school of allied Health sciences, department of Nursing and Midwifery, you and I would have a short discussion of about 20-30 minutes only and I am asking you to help us. Before we go to our discussion, I will request you to listen carefully to what I am going to read to you about the purpose and general condition of the study and you will tell me whether you agree or disagree to participate in this study at the end.

The purpose of this study is to assess the practice of key ENA messages among mothers of children birth up to two years old in Wereilu Wereda, 2018. The study will be conducted through interviews. The results of the study will inform design of the nutrition education intervention strategies targeting you. I would like to assure you that privacy will be maintained strictly throughout. A code number will identify every participant and no name will be used. Your responses to any of the questions will not be given to anyone else and no reports of the study will ever identify you. If a report of results is published, only information about the total group will appear.

The interview is voluntary and your participation / non-participation, or refusal to respond or stop responding to the questions will have no effect now or in the future on services that you or any member of your family may receive from the service providers.

Are you willing to participate in this study?

1.  [ ] Yes. 2.  [ ] No

Thank you!!!

**Annex 2. Consent form for mothers of children from birth up to two years old (English Version)**

I undersigned have been informed about the purpose of this particular research project. I have been informed that I am going to respond to this question by answering what I know concerning the issue. I have been informed that the information I give will be used only for the purpose of this study and my identity as well as the information I give will be treated confidentially. I have also been informed that I can refuse to participate in the study or not to respond to questions if I am not interested. Furthermore I have been informed that I can stop responding to the questions at any time in the process. Based on the above information I agree to participate in this research voluntarily.

Signature: ___________________

Date: ___________________

Interviewer signature certifying that informed consent has been given verbally by the respondent.

Name _________________

Signature ______________

Date __________________

Tele. _________________

3. If there are things that require clarification please don’t hesitate to ask the Interviewer or the principal investigator for clarification.

Address of the principal investigator

Biruk Beletew

Addis Ababa University, College of Health Sciences, School of allied health sciences,

Department of Nursing and Midwifery

Mobile: 09-22-89-80-70

E-mail: birukkelemb@gmail.com

Addis Ababa

**Annex 3.English version Questionnaire**

1. **Socio-demographic/economic characteristics**

| S.no | Question | Response | Skip |
| --- | --- | --- | --- |
| Background of the mother/care taker | | | |
| 101 | Age | 1. <20 years 2. 20-24 years 3. 25-29 years 4. 30-34 years 5. 35 years and above |  |
| 102 | Ethnicity | 1. Amhara 2. Oromo 3. Tigre 4. If others specify _______________ |  |
| 103 | Religion | 1. Orthodox 2. Protestant 3. Muslim 4. Catholic 5. If others specify ____________ |  |
| 104 | Marital status | 1. Single 2. Married 3. Separated 4. Divorced 5. Widowed |  |
| 105 | Educational status of the mother | 1. Cannot read and write 2. Can Read and write 3. Primary 4. Secondary 5. Higher education |  |
| 106 | Educational status of the father | 1. Cannot read and write 2. Can Read and write 3. Primary 4. Secondary 5. Higher education |  |
| 107 | Occupation of the mother | 1. Non employed 2. Housewife 3. Employed in government institution 4. Employed in private sector 5. Merchant 6. If others specify _________________ |  |
| 108 | Residence | 1. Urban 2. Rural |  |
| 109 | Monthly income | **----------------**ETB |  |
| 110 | Number of family member | 1. < 4 2. 4 – 5 3. 6 – 8 4. ≥9 |  |
| 111 | Parity(no of birth) | 1. Multipara 2. Primipara |  |
| **Back ground of the child** | | | |
| 112 | Age | 1. 0-6month 2. 7-12month 3. 13-18month 4. 19-24month |  |
| 113 | Sex | 1. Male        2. Female |  |
| **MCH service utilization** | | | |
| 114 | Where was the child born? | 1. Home      2. Institution |  |
| 115 | Did you have ANC follow up | 1. Yes          2. No |  |
| 116 | Did you have PNC service | 1. Yes           2. No |  |

1. Questionnaire on the practice Key ENA messages mother’s of children from birth up to 2 yrs  old; adapted from WHO guidelines and questionnaire (5, 73).
   1. **exclusive breast feeding**

**Practice of exclusive breast feeding**

1. Have you ever breastfed [NAME]?
2. Yes     2. No

Skip to 103

1. When did you start breast feed after birth? If less than 1 hour , record immediately(“00” hours),If less than 24 hours, record hours ,Otherwise, record days
2. Immediately
3. Hours
4. Days
5. Don’t Know
6. Did you gave sugar water, water, butter, before breast during the first days of the baby’s life?
7. Yes    2. No
8. Did you squeeze out and throw away the first milk (colostrum)?
   - 1. Yes    2. No
9. Did your baby drink anything from a bottle between sunrise yesterday and sunrise today?
10. Yes    2. No
11. **Complimentary feeding**

**Practice of complimentary feeding**

1. Did you introduce liquids or foods (semi-solid or solid) other than breast milk to the baby?
2. Yes    2. No

Skip to 205

1. At what age did you first introduce?
2. 6 months
3. 1 year
4. 2 year
5. Others
6. How many times did you feed your child solid and/or semi-solid food between sunrise yesterday and sunrise today? If response is not numeric, probe for a numeric response
7. Number of feedings of solids and/or semi-solid foods _________
8. Don’t know
9. Next I would like to ask you about some liquids and food that (NAME) may have had yesterday during the day or at night

| Group | **Food lists** | **No** | **Yes** |
| --- | --- | --- | --- |
| **Group 1**: Grains, roots and tubers | Porridge, bread, rice, noodles or other foods made from grains |  |  |
|  | White potatoes, white yams, manioc, cassava or any other foods made from roots |  |  |
| **Group 2**: Legumes and nuts | Any foods made from beans, peas, lentils, nuts or seeds |  |  |
| **Group 3**:  Dairy products | Infant formula, such as **[insert local examples**] |  | How many times? \|___\|\|___\| |
|  | Milk, such as tinned, powdered or fresh animal milk |  | How many times? \|___\|\|___\| |
|  | Yogurt or drinking yogurt |  | How many times? \|___\|\|___\| |
|  | Cheese or other dairy products |  |  |
| **Group 4**:  Flesh foods | Liver, kidney, heart or other organ meats |  |  |
|  | Any meat, such as beef, pork, lamb, goat, chicken or duck |  |  |
|  | Fresh or dried fish, shellfish or seafood |  |  |
|  | Grubs, snails or insects |  |  |
| **Group 5**:  Eggs | Eggs |  |  |
| **Group 6**:  Vitamin A fruits and vegetables | Pumpkin, carrots, squash or sweet potatoes that are yellow or orange inside |  |  |
|  | Any dark green vegetables [**insert local examples**] |  |  |
|  | Ripe mangoes (fresh or dried [not green]), ripe papayas (fresh or dried), musk melon **[insert other local vitamin-A-rich fruits]** |  |  |
|  | Foods made with red palm oil, red palm nut or red palm nut pulp sauce |  |  |
| **Group 7**:  Other fruits and vegetables | Any other fruits or vegetables |  |  |

1. Was your child breastfed or did he or she consume breastmilk yesterday during the day or at night?
2. Yes          2. No           3. Don’t know/no answer
3. **feeding of sick child during and after  illness**

**Practice Feeding of the Sick Child during and after illness**

1. Did your child was sick with in the last two weeks?
2. Yes        2. No

Skip to 305

1. How much did you breastfeed your child during and after illness? compared with the usual

1. More
2. Same as usual
3. Less than usual
4. Never gave food
5. Don’t feed
6. What amount of fluid did you offered to your child during and after illness? compared with the usual
7. More than the usual
8. Same as usual
9. Less than usual
10. Don’t give fluid
11. What amount of food did you offered to your child during and after illness? compared with the usual
12. More than usual
13. Same as usual
14. Less than usual
15. Don’t feed

**4 Nutrition of Pregnant and lactating Women**

**Practice of mothers nutrition during pregnancy and lactation**

1. Did you eat one additional meal every day during pregnancy and lactation?
2. Yes        2. No
3. Did you eat a variety of foods, particularly animal products (meat, milk, eggs, etc), plus fruits & vegetables during pregnancy and lactation?
4. Yes          2. No
5. Did you get iron/folic acid pills to maintain your strength & health during the pregnancy?   Show her or tell the color
6. Yes        2. No   3. I don’t know

**5 prevention of Vitamin A deficiency**

**Practice of mothers on prevention of Vitamin A deficiency**

1. Did you ( Mother), take Vitamin A supplementation as soon as possible (within 45 days) after delivery ?
2. Yes      2. No
3. Did your child get Vitamin A supplementation two times a year after 6 month of age?
4. Yes          2. No
5. I would like to ask you about particular foods you may eat on their own or as part of a dish.

Yesterday, during the day and night, did you eat any of the following foods?

1. Animal-source foods-Liver,Kidney, Heart,Egg yolks/egg from chicken, duck, guinea fowl or other bird,Milk, cheese, yogurt or other dairy product
2. Green vegetables-Amaranths, spinach, cassava leaves, kale and other green leafy vegetables
3. Fruits-Ripe mango, Ripe papaya, Cantaloupe,Apricot
4. I didn’t eat

**6 Prevention of anemia among mothers and children**

**Practice of mothers on prevention of anemia among mothers and children**

1. Did you (Mothers of infants 0–5 mo) received iron–folic acid during pregnancy?
2. Yes          2. No
3. Did you eat meat (liver, kidney and heart) and animal product during pregnancy?
4. Yes            2. No
5. Did you eat green leafy vegetables and fruits during pregnancy?
6. Yes            2. No
7. **Iodine**

**Practice of mothers on prevention of iodine deficiency**

1. What kind of salt did you use while you cook family food? (*If possible, ask the respondent to show you the salt.*)
2. Iodized     2. Not iodized    3. Don’t know/no answer
3. When did you add salt in to the stew?
4. At the end      2. At the middle    3. At the beginning
5. Did you store salt in dark closed container?
6. Yes                2. No
7. **Nutritional information**
8. Did you hear a message about maternal, infant and young children feeding?
9. Yes      2. No   3. Can’t remember

Skip to

1. From whom did you hear these messages?
2. Nurse
3. Doctor
4. Health extension worker
5. Radio/TV
6. Other (specify) _____________
7. When did you hear these messages? During:
8. Pregnancy
9. Delivery
10. Post natal
11. Sick child contacts
12. Well child contacts
13. Immunizations
14. Other (specify) _______________
15. Where did you hear these messages?

1. Health facility
2. Community event
3. Home
4. Other (specify) ________________

**Knowledge of exclusive breast feeding**

1. How long after birth a baby should start breastfeeding? If it less than an hour, circle immediately.
2. Immediately       2. Hours 3. Days       4. Don’t Know
3. For how long a baby should receive only breast milk and nothing else?
4. From birth to six months           2. Other 3. Don’t know
5. Babies should be given sugar water, water, butter, before first breast milk during the first days of the baby’s life?
6. Yes    2. No
7. The first yellowish milk/colostrum should be fed to the baby?
8. Yes    2. No
9. Babies should feed with which one?
10. Bottle
11. Cup
12. I don’t know

**Knowledge of complimentary feeding**

1. How long after birth a baby should start to receive semi-solid and solid foods?

Age in Months ______________

1. At six months     2. Other 3.Don’t know
2. Until what age is it recommended that a mother continues breastfeeding?
3. Six months or less
4. 6–11 months
5. 12–23 months
6. 24 months and more (correct response)
7. Other
8. How many times the child should feed solid and/or semi-solid food between per day? If response is not numeric, probe for a numeric response
9. Number of feedings of solids and/or semi-solid foods _________
10. Don’t know
11. Which one of the following food type your child should get per day?
12. Animal-source foods (meat, poultry, fish, liver/organ meat, eggs, etc.)
13. Pulses and nuts: flours of groundnut and other legumes (peas, beans, lentils, etc.), sunflower seed, peanuts, soybeans
14. Vitamin-A-rich fruits and vegetables (carrot, orange-fleshed sweet potato, mango, papaya, etc.)
15. Green leafy vegetables (e.g. spinach)
16. Energy-rich foods (e.g. oil, butter/ghee)
17. Other
18. Don’t know
19. Until what age is it recommended that a mother continues breastfeeding?
20. Six months or less
21. 6–11 months
22. 12–23 months
23. 24 months and more (correct response)
24. Other
25. Don’t know

**Knowledge of complimentary feeding**

1. How long after birth a baby should start to receive semi-solid and solid foods?

Age in Months ______________

1. At six months     2. Other 3.Don’t know
2. Until what age is it recommended that a mother continues breastfeeding?
3. Six months or less
4. 6–11 months
5. 12–23 months
6. 24 months and more (correct response)
7. Other

1. How many times the child should feed solid and/or semi-solid food between per day? If response is not numeric, probe for a numeric response
2. Number of feedings of solids and/or semi-solid foods _________
3. Don’t know
4. Which one of the following food type your child should get per day?
5. Animal-source foods (meat, poultry, fish, liver/organ meat, eggs, etc.)
6. Pulses and nuts: flours of groundnut and other legumes (peas, beans, lentils, etc.), sunflower seed, peanuts, soybeans
7. Vitamin-A-rich fruits and vegetables (carrot, orange-fleshed sweet potato, mango, papaya, etc.)
8. Green leafy vegetables (e.g. spinach)
9. Energy-rich foods (e.g. oil, butter/ghee)
10. Other
11. Don’t know
12. Until what age is it recommended that a mother continues breastfeeding?
13. Six months or less
14. 6–11 months
15. 12–23 months
16. 24 months and more (correct response)
17. Other
18. Don’t know

**Knowledge towards feeding of sick child**

1. The frequency of breast feeding during and after illness of your baby should be :
2. More than the usual
3. Same as usual
4. Less than usual
5. Don’t know
6. The amount of fluid offered for sick child during illness should be:
7. More than the usual
8. Same as usual
9. Less than usual
10. Don’t know
11. The amount of food offered to sick child during and after illness should be:
12. More than the usual
13. Same as usual
14. Less than usual
15. Don’t know

**Knowledge of mothers on nutrition during pregnancy and lactation**

1. How should a pregnant and lactating woman eat in comparison with a non-lactating woman?
2. Eat more frequently
3. Eat more protein-rich foods
4. Eat more iron-rich foods
5. Use iodized salt when preparing meals
6. Other
7. Don’t know

Most women would benefit from two types of supplements, or tablets, during pregnancy. Which are they?

- - 1. Iron supplements
    2. Folic acid supplements
    3. Other
    4. Don’t know

1. What is the health benefit for taking folic acid supplements/tablets?
2. For normal development of the nervous system of the unborn baby (brain, spine and skull)
3. To prevent birth defects/abnormalities the nervous system of the unborn baby (brain, spine and skull)
4. Other
5. Don’t know

**Knowledge of mothers on vitamin A deficiency**

1. How can one prevent a lack of vitamin A in the body?
2. Eat/feed vitamin-A-rich foods – having/giving a diet rich in vitamin A
3. Eat/feed foods fortified with vitamin A
4. Give vitamin A supplements/sprinkles
5. Other
6. Don’t know
7. Mothers should take Vitamin A supplementation as soon as possible (within 45 days) after delivery?
8. Yes         2. No 3. I don’t know
9. Can you tell me how you can recognize someone who lacks vitamin A in his or her body?
10. Weakness/feels less energetic
11. Be more likely to become sick (less immunity to infections)
12. Eye problems: night blindness (inability to see at dusk and in dim light), dry eyes, corneal damage, blindness
13. Other
14. Don’t know
15. Child should take Vitamin A supplementation two times a year after 6 month of age?
16. Yes        2. No   3. I don’t know

**Knowledge of mothers on vitamin A deficiency**

1. How can one prevent a lack of vitamin A in the body?
2. Eat/feed vitamin-A-rich foods – having/giving a diet rich in vitamin A
3. Eat/feed foods fortified with vitamin A
4. Give vitamin A supplements/sprinkles
5. Other
6. Don’t know
7. Mothers should take Vitamin A supplementation as soon as possible (within 45 days) after delivery?
8. Yes         2. No 3. I don’t know
9. Can you tell me how you can recognize someone who lacks vitamin A in his or her body?
10. Weakness/feels less energetic
11. Be more likely to become sick (less immunity to infections)
12. Eye problems: night blindness (inability to see at dusk and in dim light), dry eyes, corneal damage, blindness
13. Other
14. Don’t know
15. Child should take Vitamin A supplementation two times a year after 6 month of age?
16. Yes        2. No   3. I don’t know

**Knowledge of mothers on prevention of iron deficiency anaemia**

1. Pregnant and lactating mothers should receive iron–folic acid?
2. Yes      2. No
3. What are the health risks for infants and young children of a lack of iron in the diet?
4. Delay of mental and physical development   2. Other 3. Don’t know
5. Consumption of meat and animal product during pregnancy is helpful to prevent anemia?
6. Yes             2. No
7. Consumption of green leafy vegetables and fruits should be consumed to prevent iron deficiency anemia?
8. Yes             2. No

**Knowledge of mothers on prevention of iodine deficiency**

1. How can iodine deficiency be prevented?
2. Eat/prepare foods with iodized salt       2. Other 3. Don’t know
3. Do you know goiter can be caused by iodine deficiency
4. Yes        2. No
5. When do you think salt should be added in to a stew?
   - - 1. At the end      2. At the middle      3. At the beginning
6. Salt hould be stored in dark closed container?
7. Ye       2. No

**Attitude towards exclusive breast feeding**

1. How good do you think it is to breastfeed your baby exclusively for six months?

1. Not good                2. You’re not sure        3. Good   

1. How good do you think it is to give your child anything from bottle?

1. Not good              2. You’re not sure         3. Good

**Attitude towards complimentary feeding**

1. How good do you think it is to give different types of food to your child each day?

1. Not good             2. You’re not sure 3.  Good

1. How good do you think it is to feed your child several times each day?

1. Not good        2. You’re not sure         3. Good

1. How good do you think it is to continue breastfeeding until 24 months?

1. Not good   2. You’re not sure     3. Good

**Attitude towards feeding sick child**

1. How good do you think increasing frequency of breast feeding for your child during and after illness
2. Not good         2. You’re not sure      3. Good
3. How good do you think increasing amount fluid offered for your sick child during and after illness
4. Not good      2. You’re not sure       3. Good
5. How good do you think increasing amount food offered for your sick child during and after illness
   - - 1. Not good     2. You’re not sure     3. Good

**Attitudes of mothers towards nutrition during pregnancy and lactation**

1. How good do you think it is to eat more food during pregnancy?

1. Not good     2. You’re not sure      3. Good

1. How serious do you think iron-deficiency/anaemia is?

1. Not serious      2. You’re not sure     3. Serious

1. How good do you think it is to prepare meals with iron-rich foods such as beef, chicken or liver?

 1. Not good       2. You’re not sure    3. Good

**Attitude of mothers towards vitamin A deficiency**

1. How serious do you think a lack of vitamin A is?

1. Not serious        2. You’re not sure   3. Serious

1. How good do you think it is to prepare meals with vitamin-A-rich foods such as carrots, green leafy vegetables, sweet-potatoes or liver?

1. Not good         2. You’re not sure        3. Good

**Attitude of mothers towards prevention of anemia among mothers and children**

1. How likely do you think you are to be iron-deficient/anaemic?
   - - 1. Not likely     2. You’re not sure        3. Likely
2. How serious do you think iron-deficiency/anaemia is?
   - - 1. Not serious    2. You’re not sure    3. Serious
3. How good do you think it is to prepare meals with iron-rich foods such as beef, or liver?

 1. Not good    2. You’re not sure     3. Good

**Attitudes of mother towards iodine deficiency**

1. How serious do you think a lack of iodine in the body is?

1. Not serious    2. You’re not sure    3. Serious

1. How good do you think it is to prepare meals with iodized salt?

1. Not good     2. You’re not sure         3. Good

Annex 4: Amharic Version Information sheet

**እድሚያቸዉ ከውልደት እስከ 24 ወራት ባሉ ህፃናት እናቶች የመረጃ መስጫና የፈቃደኝነት መጠየቂያ ቅጽ**

**አድስ አበባ ዩኒቨርሲቲ**

**ጤናሳይንስ ኮሌጅ፣ ነርሴንግና ሜድዋይፈሪ ት/ት ክፍል**

**ክፍልአንድ :የመረጃመስጫቅጽ**

**መግቢያ**፡እንደምንአደሩ / ዋሉ ስሜ------------------------ ይባላል፡፡በአድስ አበባ ዩኒቨርስቲ ፣ በጤና ሳይንስ ፋኩሉቲ ፤ በነርሲንግና ሚዴዋይፈሪ ት/ቤት አስተባባሪነት በሚከናወነው ጥናት እኔና እርሰዎ አጠር ያለ ፣ ከ 20-30 ደቂቃ የሚወስድ ውይይት ይኖረናል ፡፡ ለዚህም ውይይት እንድተባበሩኝ በትህትና እጠይቃሇሁ ፡፡ ወደ ውይይቱ ከመግባታችን በፊት ስለጥናቱ አላማና ጠቅላላ ሁኔታ ስለማነብልወት በጥሞና እንዲያዳምጡን እጠይቃለሁ ፡፡ በመጨረሻም በጥናቱ ለመሳተፍ መስማማወን እና አለመስማማትወን ይነግሩኛል፡፡

የዚህ ጥናት አላማ በወረኢሉ ወረዳ የሚኖሩ እድሚያቸው ከ 2 ዓመት በታች የሆኑ ልጆች ያላቸው እናቶች ስለራሳቸው እና ልጆቻቸው አመጋገብ ያላቸውን ግንዛቤ ፣ አመለካከት እና ተግባር ምን እንደሚመስል ለማወቅ ሲሆን ጥናቱ የሚካሄድበት መንገድ በመረጃ ሰበሰሳቢው በሚቀርብ መጠይቅ ይሆናል ፡፡ እርሰዎ የሚሰጡት መረጃ በወረኢሉ እና በሀገር አቀፍ ደረጃ ለሚገኙ እናቶች እና ህፃናት ስለምግብ የሚያስፈሌጋቸውን መረጃ ለማዲረስ ይረዳል፡፡

በቆይታዎ ሁለ ምስጢር እንደምንጠብቅ እያረጋገጥኩኝ ለእያንዲነዱ ተሳታፊ የተለየመለያ ቁጥርሲሆን ስምዎን ግን አንጠቅስም ፡፡ ለማንኛውም ጥያቄ የሚሰጡት ምላሽ ለሌላ ሰው ተላልፎ አይሰጥም ፡፡ የጥናቱ ውጤት ሪፖርትም ስለእርሰዎ አይገልጽም ፡፡

በተጨማሪም የጥናቱ ሪፖርት ቢታተም የሚያወጣው ስለ አጠቃሊይ ተሳታፊሰዎች ብቻ ይሆናል ፡፡ መጠይቁ በፈቃደኝነት ላይ ብቻ ሲሆን የእርሰዎ መሳተፍ ወይም አለመሳተፍ እንዱሁም ጥያቄዎችን ለመመለስ ፈቃደኛ አለመሆንና በጥቄው ወቅት አቋርጦ መውጣት አሁንም ይሁን ወደፊት እርሰዎ ምይሁን ቤተሰበዎ በሚያገኙት አገልግሎት ላይ ምንም አይነት ተጽዕኖ አይኖረውም ፤ በጥናቱ ላይ ተሳታፊ በመሆነዎም የነሚሰጥ ክፍያም አይኖርም ፡፡ ለመሳተፍ ፈቃደኛ ነዎት?

1.  ( ) አዎ      2. ( )  አይደለሁም

አመሰግናለሁ!!!

ማስታወሻ ፡ የጥናቱ ተሳታፊ በጥናቱ ላይ ለመሳተፍ ፈቃደኛ ከሆኑ ወደ ፈቃደኝነት ማረጋገጫ ቅጽ ይለፉ፡፡

Annex 5: Amharic version consent form (ፈቃደኝነትማረጋገጫቅጽ የአማርኛ ትርጉም)

ክፍል 2፡-እድሚያቸዉ ከ 2 ዓመት በታች የሆኑ ልጆች ያላቸው እናቶች የፈቃደኝነት መጠየቂያ ቅጽ

ከታች ፊርማየን ያኖረኩት እኔ የጥናቱ ዓላማ የተነገረኝ ሲሆን የምጠየቀው ጥያቄ የማቀውን መመለስ እንደምችል ፤ እኔ የምሰጠው መረጃ ለዚህ ጥናት አገላግሎት ብቻ የሚውል ሲሆን ስሜንና የምሰጠውን መረጃ በምስጢር እንደሚጠበቅ ተነግሮኛል ፡፡ ፍላጎት ከሌለኝ በጥናቱ ያለመሳተፍ ፤ ጥያቄ ያለመመለስና በጥያቄው መካከል አቋርጨ መውጣት እንደምችል ተነግሮኛል ፡፡ በዚህ መሰረት በጥናቱ ለመሳተፍ ፈቃደኛ መሆኔን በፊርማ የአረጋግጣሁ፡፡

ፈርማ ----------------------------ቀን ------------------------------ማስታዎሻ፡

1.  የጥናቱ ተሳታፊ በጥናቱ ፈቃደኛ ከሆኑ መጠይቁን ይጀምሩ፡፡

2.  የጥናቱ ተሳታፊ ፈቃደኛ መሆናቸውን የሚያረጋግጥ የመረጃ ሰብሰሳቢው ስምና ፊርማ

ስም _________________________________________________________

ፈርማ__________________________________

ስልክ ____________________________________

ማንኛውም ገለጻ የሚያስፈልጋቸው ነገሮች ካሉ መረጃ ሰበሰሳቢውንም ሆነ ዋና ተመራማሪውን በአካልም ሆነ በአድራሻው ይጠይቁ፡፡

የዋና ተመራማሪው አድራሻ፡

ብሩክ በለጠው

አዱስ አበባ ዩኒቨርሲቲ ፤ ጤና ሳይንስ ኮሌጅ ፤ በነርሲነግና ሚዴዋይፈሪ ድህረምረቃ ት/ቤት

ስልክቁጥር- 09 22898070, birukkelemb@gmail.com , አድስ አበባ

Annex 6. Amharic Version Questionnaire

ክፍሌ 1 ፡ ማህበራዊ ፣ ነባራዊና ኢኮኖሚያዊ ሁኔታ

1. እድሜሽ/ዎት ስንት ነው?

- - - 1. <20 years
      2. 20-24 years
      3. 25-29 years
      4. 30-34 years
      5. 35 እና ከዛ በላይ

1. 35 years and above የየትኛው ብሄረሰብ አባሌ ኖት?

1. አማራ
2. ትግሬ
3. ኦሮሞ
4. ጉራጌ
5. ሌላካለ (ይጠቀስ)----------------

1. የትኛውን ሀይማኖት ተከታይ ኖት ?
2. ኦርቶዶክስ ተዋህዶ

1. ሙስሊም
2. ካቶሊክ
3. ፕሮቴስታንት
4. ሌላካለ (ይጠቀስ)----------------

1. የጋብቻ ሁኔታዎን ይግለፁልኝ

1. ያላገባ
2. ባለትዲር
3. የፈታች
4. ባሌ የሞተባት
5. የተለያየች

1. የትምህርት ደረጃ

1. ያልተማረች
2. 1-8 ኛክፍል
3. 9-12ኛ ክፍል
4. የኮሌጅ ት/ት እና ከዛ በላይ

1. የባለቤትሽ/ወየት/ትደረጃ

1. ያልተማረ
2. 1-8 ኛክፍል
3. 9-12ኛ ክፍል
4. የኮሌጅ ት/ት እና ከዚያ በላይ

1. የስራ ሁኔታ

1. የቤት እመቤት
2. የግል ስራ
3. የመንግስት ሰራተኛ
4. ሌላ ካለ (ይጠቀስ)----------------

1. የመኖሪያ ቦታ
2. ከተማ
3. ገጠር
4. ባጠቃ ላይ ምንያህል ወርሀዊ ገቢ ያገኛሉ?-----------------------ኢትዮ. ብር
5. የቤተሰብ ብዛት

1. አንድ
2. ሁለት
3. ሦስት
4. አራት
5. አምስትና ከዛ በላይ

1. ባጠቃላይ ምን ያህል ልጅ ወልደዋል? -?
   - - 1. አንድ
       2. ሁለትና ከዚያ በላይ

**ልጅን የሚመለከት**

1. ልጅሽ ስንት አመቱ / ቷ ነው?

1. 0-6 ወር
2. 7-12 ወር
3. 13-18 ወር
4. 19-24 ወር

1. የልጅሽ ፆታ
2. ወንድ        2. ሴት

አገልግሎት አጠቃቀምን በተመለከተ

1. ልጅወን የት ነው የወለዱት?
2. እቤቴ          2. ጤና መስሪያ ቤት
3. የቅድመ- ወሊድ ክትትል አድርገዋል?
4. አዎ        2. አላደረኩም
5. የድህረ- ወሊድ ክትትል አድርገዋል?
6. አዎ      2. አላደረኩም

**በቁልፍ አስፈላጊ የአመጋገብ ድርጊት መልእክቶች ላይ ከውልደት እስከ 24 ወራት እድሜ ባሉ ህፃናት እናቶች መካከል በሚኖረው እውቀት ፣ አመለካከት እና ተግባር ላይ የቀረበ መጠየቅ የአማርኛ ትርጉም**

**1 የእናት ጡት ብቻ ስለመመገብ**

**ተግበርን የተመለከተ መጠይቅ**

1. ጡት አጥብተዉ ያዉቃሉ?
2. አወ    2. አላዉቅም

መልስወ አላዉቅም ከሆነ ወደ ጥያቄ ቁጥር 103 ይሂዱ

1. ከወለዱ ከምን ያክል ጊዜ በሗላ ነበር ለልጅወ ጡት የሰጡት?

1. ወዳዉኑ        2. ከሰዓታት በሗላ        3. ከቀናት በሗላ        4. አላዉቅም

1. ለልጅወ በመጀመሪያወቹ ቀናት ዉስጥ ጡት ከመጀመራቸዉ በፊት ዉሀ በስዃር ፣ ዉሀ ወይም ቅቤ ሰጥተዉት ነበር?
2. አወ    2. አልሰጠሁም
3. የመጀመሪያዉን ቢጫ ወተት (እንገር) አልበዉያ ፈሳሉ?
4. አወ    2. አላፈስም
5. ትላንት ፀሀይ ከወጣች ጀምሮ እስከ ዛሬ ልጅወ ከጡጦ የጠጣዉ ነገር አለ?
6. አዎ    2. የለም

**አመለካከትን የተመለከተ መጠይቅ**

1. ልጅን እስከ 6 ወር ጡት ብቻ መስጠት ምን ያክል ጥሩ ነዉ ብለው ያስባሉ?
2. ጥሩ አይደለም    2. እርግጠኛ አይደለሁም    3. ጥሩ ነው
3. ለልጅ በጡጦ መመገብ ምን ያክል ጥሩ ነዉ ብለው ያስባሉ?
   - - 1. ጥሩ አይደለም    2. እርግጠኛ አይደለሁም    3. ጥሩ ነው

**ግንዛቤን የተመለከተ መጠይቅ**

1. ህፃናት ከተወለዱ ከስንት ጊዜ በሗላ ጡት መጥባት አለባቸዉ?

1. ወዳዉኑ        2. ከሰዓታት በሗላ        3.ከቀናት    በሗላ    4.አላዉ
2. ህፃናት የእናታቸዉን ጡት ብቻ ለምን ያህል ጊዜ መመገብ አለባቸዉ?
3. እስከ 6 ወርድረስ    2. ሌላካለይጠቀስ    3. አላዉቅም
4. ህፃናት በመጀመሪያወቹ ቀናት ዉስጥ ጡት ከመጀመራቸዉ በፊት ዉሀ በስዃር ፣ ዉሀ ወይም ቅቤ መሰጠት አለባቸዉ?

1. አወ        2. የለባቸዉም

1. የመጀመሪያዉን ቢጫ ወተት (እንገር) ህፃናት መመገብ አለባቸዉ?
2. አወ    2. የለባቸዉም
3. ህፃናትን በየትኛዉ መንገድ መመገብ አለብን?
4. በጡጦ    2. ስኒ    3. አላዉቅም

**2 ተጨማሪ ምግብን በተመለከተ**

**ተግባርን በተመለከተ**

1. ለልጅወ ከጡት ሌላ ፈሳሽ ፣ ጠጣር እና ለስለስ ያሉ ምግቦችን አስጀምረዉለታል?
2. አወ    2. አላስጀመርኩትም

መልስወ አላስጀመርኩትም ከሆነ ወደ ጥያቄ ቁጥር 205 ይሂዱ

1. መጀመሪያ መቼ ነበር ያስጀመሩት?

1. 6 ወር
2. 1 አመት
3. 2 አመት
4. ሌላካለ (ይጠቀስ)¬¬¬¬¬¬¬¬

1. ትላንትፀሀይከወጣችበትጊዜጀምሮእስከዛሬፀሀይእስከወጣችበትጊዜድረስልጅወንጠጣረናለስለስያሉምግቦችንስንትጊዜሰጡት?
2. በቁጥር------    2. አላዉቅም
3. ከዚህበመቀጠልበትላንትናዉእለትቀንናምሸትለልጅወጠጣርእናለስለስያሉምግቦችንሰጥተዉከሆነእጠይቅወታለሁ?

| ቡድን | የምግብ ዝርዝሮች | አወ | አይደለም |
| --- | --- | --- | --- |
| ቡድን**1**: ከጥራጥሬ ፣ ከስራስሮች እና ክብ ስር ያላቸው ተክሎች | ገንፎ ፣ ዳቦ ፣ ሩዝ ፣ ወይም ከጥራጥሬ የተሰሩ ሌሎች ምግቦች |  |  |
|  | ነጭ ድንች ፣ ነጭ የስር ተክል ፣ የካሳቫ ስር ፣ ካሳቫ ወይም ከስራስር የሚዘጋጁ ሌሎች |  |  |
| ቡድን**2**: የብርተክሎች፣የለውዝ ተክል፣ | ከአተር፣ ባቄላ ፣ምስር ፣ኦቾሎኒ ወይም ዘሮች የሚዘጋጁ ማናቸውንም ምግቦች |  |  |
| ቡድን**3**:  የወተትተዋጽኦምርቶች | የጨቅላ ህፃናት ምግብ፣ (የሀገር ውስጥ ምሳሌዎችን ያስገቡ) |  |  |
|  | ወተት፣የተናጠ፣በፓውደር መልክ ወይም ትኩስ የእንስሳት ወተት |  |  |
|  | እርጎ |  |  |
|  | አይብ ወይም ሌሎች የእንስሳት ተዋጽኦ ምርቶች |  |  |
| ቡድን**4**:  **ከስጋ**የሚዘጋጁምግቦች | ጨጓራ፣ጉላሊት፣ልብ ወይም የሌሎች የሰውነት ክፍል ስጋዎች |  |  |
|  | ማንኛውም ስጋ፣ የከብት ስጋ፣ የአሳማስጋ፣ የበግስጋ፣የፍየል፣የዶሮ ወይም የዳክዬ |  |  |
|  | ትኩስ ወይም የደረቀ አሳ፣ሼል አሳ ወይም የባህር ምግብ |  |  |
|  | ግራብ፣ቀንድ አውጣዎች ወይም በራሪ ነብሳት |  |  |
| ቡድን**5**:  እንቁላል | እንቁላል |  |  |
| ቡድን6:  ቫይታሜን ኤ ፍራፍሬወች እና ቅጠላቅጠሎች | ዱባ፣ካሮት፣ስኳሽ ወይም ስኳርድንች እንዲሁም አረንጓዴተክሎች (የአገር ውስጥ ምሳሌዎችን ያስገቡ) |  |  |
|  | የደረሰ ማንጎ (ትኩስ ወይም የደረቀ (አረንጓዴ))፣የደረሰ ፓፓዬ (ትኩስ ወይም የደረቀ)፣ሃባብ (የሌሎች የአገር ውስጥ በቪታሚንኤ የበለፀጉ ፍራፍሬዎችን ያስገቡ) |  |  |
|  | በቀይ የዘንባባ ዘይት የተሰሩ ምግቦች በቀይ የዘንባባ ፍሬ ወይም በቀይ የዘንባባ ፍሬ የተሰራ የፐልፕሶስ |  |  |
| ቡድን7:  ሌሎች አትክልት ወይም ፍራፍሬዎች | ሌሎች ፍራፍሬዎች እና አረንጓዴ ተክሎች |  |  |

1. ልጅሽ በትላንትናዉ እለት ፣ ማለትም ከጧት እስከ ማታ ጡት ጠብቷል ?
2. አወ    2. አልጠባም    3. አላዉቅም

**አመለካከትን በተመለከተ**

1. ለልጅ በቀን የተለያዩ አይነት ምግቦችን መስጠት ምያ ክልጥሩ ነው ብለው ያስባሉ?
2. ጥሩ አይደለም    2. እርግጠኛ አይደለሁም    3. ጥሩ ነው
3. ለልጅ በቀን ብዙ ጊዜ ምግቦችን መስጠት ምያክል ጥሩ ነው ብለው ያስባሉ?
4. ጥሩ አይደለም    2. እርግጠኛ አይደለሁም    3. ጥሩ ነው
5. ልጅን እስከ ሁለት አመት ድረስ ጡት ማጥባት ጥሩ ነው ብለው ያስባሉ?
   - - 1. ጥሩ አይደለም    2. እርግጠኛ አይደለሁም    3. ጥሩ ነው

**ግንዛቤን በተመለከተ**

1. ህፃናት ከተወለዱ ከስንት ጊዜ በሗላ ጠጣረና ለስለስ ያሉ ምግቦችን መጀመር አለባቸዉ?
2. በ 6 ወር    2. ሌላ    3. አላዉቅም
3. ህፃናት እስከ ስንት አመት ጡት መጥባት አለባቸዉ?

1. እስከ 6 ወር
2. ከ 6–11 ወር
3. ከ 12–23 ወር
4. 24 ወርእናከዚበላይ
5. ሌላ

1. ህፃናት ለስለስ ያሉ ምግቦችን በቀን ስንት ጊዜ መመገብ አለባቸዉ ብለዉ ያስባሉ?
2. በቁጥር------    2. አላዉቅም
3. ከሚከተሉት ምግቦች መካከል ልጅሽ በቀን የትኞቹን ማግኘት አለበት?
4. የእንስሳት ተዋፅዖ ምግቦች እንደ ፤ ስጋ ፣ የወፍ ስጋ ፣ ጉበት ፣ እንቁላል እና የመሳሰሉትን
5. ለውዝ: የኦቾሎኒ ዱቄት እና ሌሎች ጥራጥሬወች እንደ ፤ አተር ፣ባቄላ እና የመሳሰሉትን
6. በቫይታሚን ኤ የበለፀጉ ፍራፍሬ እና አትክልት ምግቦች እንደ (ካሮት ፣ ብርቱካን ፣ ድንች ፣ ማንጎ ፣ ፓፓያ ፣ እናየመሳሰሉትን
7. አረንጏዴ ቅጠላ ቅጠልአማ ምግቦች ለምሳሌ ጎመን
8. ሀይል ሰጭ ለምሳሌ ዘይት, ቅቤ
9. ሌላ
10. አላዉቅም
11. እናቶች ልጆቻቸዉን እስከ መቸ እንድያጠቡ ይመከራል?

1. ወር እና ከዚያ በታች
2. ከ 6-11  ወር
3. ከ 12-23 ወር
4. 24 ወር እናከ ዚያ በላይ
5. ሌላ
6. አላዉቅም

3 **የታመመ ህፃንን አመጋገብ በተመለከተ**

**ተግባርን በተመለከተ**

1. ባለፉት 2 ሳምንታት ልጅወ ታሞብወት/ ማብወት ነበር?
2. አወ
3. አልታመመብኝም/ችብኝም
4. መልስወ አልታመመብኝም/ችብኝም ከሆነ ወደጥያቄ ቁጥር 305 ይሂዱ
5. ህፃኑ በታመመ ጊዜ ለምን ያክል ጊዜ አጠባሽዉ ፣ ከወትሮዉ ስታወዳድሪ?

1. ከወትሮዉ በበለጠ
2. ልክ እንደ ወትሮዉ
3. ከወትሮዉ ባነሰ
4. አላጠባም

1. አላዉቅም
2. ህፃኑ በታመመ ጊዜ ምን ያክል ፈሳሽ ስጠሽዉ ፣ ከወትሮዉ አወዳድረሽ?

1. ከወትሮዉ በበለጠ
2. ልክ እንደ ወትሮዉ
3. ከወትሮዉ ባነሰ
4. ፈሳሽ አልሰጥም

1. ህፃኑ በታመመ ጊዜ ምን ያክል ምግብ ስጠሽዉ ፣ ከወትሮዉ አወዳድረሽ?

1. ከወትሮዉ በበለጠ
2. ልክ እን ደወትሮዉ
3. ከወትሮዉባነሰ
4. ምግብ አልሰጠውም

**አመለካከትን በተመለከተ**

1. ልጆች ሲታመሙ ከበፊቱ በተለየ ቶሎ ቶሎ መመገብ ምን ያክል ጥሩ ነው ብለው ያስባሉ?
2. ጥሩ አይደለም    2. እርግጠኛ አይደለሁም    3. ጥሩነው
3. ልጆች ሲታመሙ ከበፊቱ በተለየ የሚመገቡትን ምግብ መጨመር ምን ያክል ጥሩ ነው ብለው ያስባሉ?
   - - 1. ጥሩ አይደለም    2. እርግጠኛ አይደለሁም    3. ጥሩነው
4. ልጆች ሲታመሙ ከበፊቱ በተለየ የሚጠቡትን ጡት መጨመር ምን ያክል ጥሩ ነው ብለው ያስባሉ?
   - - 1. ጥሩ አይደለም    2. እርግጠኛ አይደለሁም    3. ጥሩነው

**ግንዛቤን የተመለከ ተመጠይቅ**

1. ህፃናት በታመሙ ጊዜ ለምን ያክል ጊዜ መጥባት አለባቸዉ?

1. ከወትሮዉ በበለጠ
2. ልክ እንደወትሮዉ
3. ከወትሮዉ ባነሰ
4. አላወቅም

1. ህፃናት በታመሙ ጊዜ ምን ያክል ፈሳሽ መዉሰድ አለባቸዉ?

1. ከወትሮ ዉበበለጠ
2. ልክ እንደ ወትሮዉ
3. ከወትሮዉ ባነሰ
4. አላወቅም

1. ህፃናት በታመሙ ጊዜ ምን ያክል ምግብ መዉሰድ አለባቸዉ?

1. ከወትሮዉ በበለጠ
2. ልክ እንደ ወትሮዉ
3. ከወትሮ ዉባነሰ
4. አላወቅም

**4 የነፍሰጡር እና አጥቢ እናቶች አመጋገብ**

**ተግባርን በተመለከተ**

1. ነፍሰጡር እያለሽ እና ስታጠቢ ከበፊቱ በበለጠ ተጨማሪ ቢያንስ አንድ ጊዜ ትመገቢ ነበር?
2. አዎ    2. አልመገብም
3. ነፍሰጡር እያለሽ እና ስታጠቢ ከተለያዩ የምግብ አይነቶች በተለይ ስጋ ፣ ወተት ፣ እንቁላል ፣ የተለያዩ ፍራግሬወች እና ቅጠላቅጠሎችን ትመገቢ ነበር?
4. አዎ    2. አልመገብም
5. ነፍሰጡር እያለሽ እና ስታጠቢ ቫይታሚን ቢ9 (ፎሉክ አሲዴ) ተሰቶሽ ነበር? (አሳይ ወይም ቀለሙን መናገር )
6. አዎ    2. አልተሰጠኝም    3. አላውቅም

**አመለካከትን በተመለከተ**

1. በእርግዝና ጊዜ ከበፊቱ በተለየ ብዙ ምግብ መመግብ ምን ጥሩ ነው ብለው ያስባሉ?
2. ጥሩ አይደለም    2. እርግጠኛ አይደለሁም    3.  ጥሩ ነው
3. በአይረን እጥረት የሚመጣ ደም ማነስ ምንያክል ከባድ ነው ብለው ያስባሉ?
4. ከባድ አይደለም    2. እርግጠኛ አይደለሁም    3. ከባድ ነው
5. በእርግዝና ጊዜ በአይረን የበለዐጉ ምግቦችን ማዘጋጀት ጥሩ ነው ብለው ያስባሉ?
6. ጥሩ አይደለም    2. እርግጠኛ አይደለሁም    3. ጥሩ ነው

**ግንዛቤን የተመለከተ መጠይቅ**

1. ነፍሰ ጡር እና አጥቢ እናቶች ከማያጠቡትጋር ስናወዳድር እንደት መመገብ አለባቸዉ?

1. ከወትሮዉ በበለጠ
2. ገንቢ ምግቦችን በበለጠ ሁኔታ መመገብ አለባቸዉ
3. በአይረን የበለዐጉ ምግቦችን መመገብ አለባቸዉ
4. ምግብን ስናዘጋጅ በአዮድን በበለገ ጨዉ መሆን አለበት
5. ሌላ
6. አላዉቅም

1. ብዙ እናቶች በእርግዝና ጊዜ በሚሰጡ ሁለት መድሀኒቶች ይጠቀማሉ ፣ እነሱም, (አሳይ ወይም ቀለሙን መናገር )
2. አይረን    2. ፎሊክአሲድ    3. ሌላ       4. አላዉቅም
3. በእርግዝና ጊዜ ፎሊክ አሲድ መውሰድ ለምን ይጠቅማል?
   - - 1. ጤንማ የአእምሮ እድገት እንድኖር ያደርጋል
       2. የወሊድ ጊዜ እክሎችን / ችግሮችን ፣ ያልተወለደን ህፃን የነርቭ ስርአት (አንጎል ፣ ህብረሰረሰር እና እራስቅል) ችግሮች ለመከላከል
       3. ሌላ
       4. አላዉቅም

**5 ቫይታሚን ኤን የተመለከተ**

**ተግባርን በተመለከተ**

1. ከወለድሽበ 45 ቀን ዉስጥ ቫይታሚን ኤ ወስደሻል?
2. አወ    2. አልወሰድኩም    3. አላዉቅም
3. ልጅ] ከተወለደ ከ 6 ወር በሓላ በአመት ሁለት ጊዜ ቫይታሚን ኤ ወስዳል?
4. አወ    2. አልወሰደም    3. አላዉቅም
5. ከዚህ በመቀጠል በትላንትናዉ እለት ቀንና ምሸት ሊመገቧቸው የሚችሏቸውን ዝርዝር ምግቦችን በተመለከተ ጥያቄል ጠይቆት እወዳለሁ፡፡ትላንትና ቀን ላይ እና ምሽት ከሚከተሉት የትኛውን ተመግበዋል?
6. **የእንስሳት ምንጭ**- ጨጓራ ፣ ኩላሊት ፣ ልብ ፣ የእንቁላል አስኳል/የዶሮ እንቁላል ፣የ ዳክዬ ፣የ ጊኒ ወፍ ወይም ሌላ ወፍ ፣ ወተት ፣ አይብ ፣እርጎ ወይም ሌሎች የወተት ተዋጽኦ ምርቶች
7. **አረንጓዴ አትክልቶች-** አማራንትስ ፣ ስፒናች ፣ ካሳቫ ቅጠሎች፣ ጥቅል ጎመን እና ሌሎች አንጓዴ ቅጠሎች ያላቸው አትክልቶች
8. **ፍራፍሬዎች**- የደረሰ ማንጎ ፣ የደረሰ ፓፓዬ ፣ መንደሪን ፣ አፕሪኮት
9. አልበላሁም

**አመለካትን በተመለከተ**

1. የቫይታሚን ኤ እጥረት ምን ያክል ከባድ ነው ብለው ያስባሉ?
2. ከባድ አይደለም    2. እርግጠኛ አይደለሁም    3. ከባድ ነው
3. ምግብ ሲዘጋጅ በቫይታሚን ኤ በበለዐጉ ምግቦች እንደ ፣ ካሮት ፣ አርንጋዴ ቅጠል ያላቸው አትክልቶች ፤ ጣፋጭ ድንች እና ጉበት መጨመር ምን ያክል ጥሩ ነው ብለው ያስባሉ?
4. ጥሩ አይደለም    2. እርግጠኛ አይደለሁም    3. ጥሩ ነው

**ግንዛቤን የተመለከተ መጠይቅ**

1. አንድ ሰው የቪታሚን ኤ እጥረት በሰውነቱ ውስጥ በምን መልኩ ሊከላከል ይችላል?
2. በቪታሚን ኤ የበለፀጉ ምግቦችን
3. በቪታሚን ኤ የተሞሉ ምግቦችን ይመገቡ
4. በቪታሚን ኤ የታከሉ
5. የቪታሚን ኤ ጭማሪዎችን የሚሰጡ ምግቦችን ይመገቡ
6. ሌላ
7. አላውቅም
8. እናቶች እንደወለዱ ወዲያዉኑ ቫይታሚን ኤ ማግኘት አለባቸዉ?
9. አወ    2. የለባቸዉም    3. አላዉቅም
10. የቫይታሚን ኤ እጥረት ያለባቸዉ ሰወች የሚያሳዩትን ምልክቶቸ ልትነግሪኝ ትችያለሽ?
11. ድካም እና አቅም ማነስ
12. የመታመም እድል አላቸዉ
13. የአይን ችግር ፣ ማታ ማታ የማየት ችግር ፣ የአይን መድረቅ ፣ ማየት መሳን
14. ሌላ
15. አላዉቅም
16. ህፃናቶች ከ 6 ወር በኋላ ቫይታሚን ኤ ባመት 2 ጊዜ ማግት አለባቸዉ ብለሽታ ስቢያለሽ?
17. አወ    2. አላስብም    3. አላዉቅም

**6 ደም ማነስን ስለመከላከል**

**ተግባርን በተመለከተ**

1. ነፍሰጡር እያለሽ አይረን እና ፎሊክ አሲድ ነበር?
2. አዎ    2. አልወሰብም    3. አላዉቅም
3. ነፍሰጡር እያለሽ ስጋ እና የእንስሳት ተዋፅኦ ትመገሚ ነበር?
4. አዎ    2. አልወሰብም    3. አላዉቅም
5. በእርግዝናጊዜአረንጓደቅጠላቅጠሎችንእናፍራፍሬወችንትመገሚነበር?
6. አዎ    2. አልወሰብም    3. አላዉቅም

**አመለካከትን በተመለከተ**

1. በአይረን እጥረት ምክናየት በሚመጣ ደም ማነስ የመያዝ እድል አለኝ ብለው ያስባሉ?
2. የመያዝ እድል አለኝ    2. እርግጠኛ አይደለሁም    3. የመያዝ እድል አለኝ
3. በአይረን  እጥረት ምክናየት የሚመጣ ደም ማነስ ምን ያክል ከባድ ነው ብለው ያስባሉ?
4. ከባድ አይደለም    2. እርግጠኛ አይደለሁም    3. ከባድ ነው
5. ምግብ ሲዘጋጅ በአይረን በበለዐጉ ምግቦች እንደ የከብት ስጋ ፣ ጉበት ተጨምሮ ቢዘጋጅ ጥሩ ነው ብለው ያስባሉ?
6. ጥሩ አይደለም    2. እርግጠኛ አይደለሁም    3. ጥሩ ነው

**ግንዛቤን በተመለከተ**

1. ነፍሰጡር እና አጥቢ እናቶች አይረን እና ፎሊክ አሲድ መወሰድ አለባቸዉ
2. አዎ    2. የለባቸውም    3. አላዉቅም
3. ህዓናት በሚመገቡት ምግብ ውስጥ የአይረን እጥረት ካለውምን የጤና እክል ይገጥማቸዋል?
4. የአእምሮ እና አካላዊ እድገት መዘግየት    2. ሌላ    3. አላዉቅም
5. ስጋን እና የእንስሳት ተዋፅኦ በእርግዝና ጊዜ መመገብ ደም ማነስን ይከላከላል ብለዉ ያስባሉ?
6. አዎ    2. አላስብም    3. አላዉቅም
7. አረንጓደ ቅጠላቅጠሎችን እናፍራፍሬወችን በእርግዝና ጊዜ መመገብ አለብን
8. አዎ    2. የለብንም    3. አላዉቅም

**7 አዮድንን በተመለከተ**

**ተግባር**

1. የቤተሰብ ምግብ ስታዘጋጅ የትኛዉን አይነት ጨዉ ትጠቀሚያለሽ?
2. በአዮድን የበለፀገ ጨዉ    2. በአዮድን ያልበለፀገ ጨዉ    3. አላዉቅም
3. የቤተሰብ ምግብ ስታዘጋጅ ጨዉ መቸ ነዉ የምትጨምሪዉ?
4. መጨረሻ        2. መካከል    3. መጀመሪያ
5. ጨዉ የምታስቀምጭዉ የተከደነ እቃ ዉስጥ ነዉ?
6. አዎ    2. አይደለምም    3. አላዉቅም

**አመለካከትን በተመለከተ**

1. በሰውነታችን ውስጥ የአዮድን እጥረት መኖር ከባድ ነው ብለው ያስባሉ?
2. ከባድ አይደለም    2. እርግጠኛ አይደለሁም    3. ከባድ ነው
3. ምግብን በአዮድን በበለዐገ ጨው ማዘጋጀት ጥሩ ነው ብለው ያስባሉ?
4. ጥሩ አይደለም    2. እርግጠኛ አይደለሁም    3. ጥሩ ነው

**ግንዛቤን በተመለከተ**

1. የአዮድን እጥረትን እንደት መከላለል እንችላለን?
2. በአዮድን የበለዐገ ምግብ መመገብ    2. ሌላ    3. አላውቅም
3. እንቅርት በአዮድን እጥረት ሊመጣይችላል?
4. አዎ    2. አያመጣምም    3. አላዉቅም
5. ምግብ ስናዘጋጅ ጨዉ መጨመር ያለብን መቸ ነዉ?
6. መጨረሻ        2. መካከል    3. መጀመሪያ
7. ጨዉማስቀመጥያለብንየተደነእቃዉስጥነዉ?
8. አዎ    2. አይደለምም    3. አላዉቅም
   1. **ስለ ምግብ ያለወትን መረጃ በተመለከተ**
9. ስለ እናቶችና ህዓንት ስርዓተ-ምግብ መረጃ አግኝተዋል?
10. አዎ    2. አላገኘሁም    3. አላስታዉስም
11. መልስዎ አዎ ከሆነ ፡መረጃውን ከየት አገኙ?

1. ነርስ
2. ዶክተር
3. ጤና ኤክስቴንሽን ሰራተኛ
4. ከጓደኛ
5. ሌሎችምንጮች____________________
6. መረጃዉን መቸ ሰማሽ?

1. በእርግዝና ጊዜ
2. በወሊድ ጊዜ
3. ድህረ -ወሊድ
4. ህፃን ሳሳክም
5. ጤናማ ህፃን  ክትትል
6. በክትባት ጊዜ
7. ሌላ ካለ ይጠቀስ--------------

1. መረጃዉን የት ሰማሽ?
2. ጤና መስሪያ ቤት
3. ማህበረሰብ
4. ከቤት
5. ሌላ ካለ ይጠቀስ--------
